# Upstream CB1R Regulation of β‐Adrenergic Memory Consolidation

**DOI:** 10.1002/hipo.70084

**Published:** 2026-02-13

**Authors:** Janine I. Rossato, Lucas Ribeiro, Thais Lima‐Silva, Raquel Araujo, Rodrigo Orvate, Anna Lyvia Azevedo, Nina Bevilaqua Cammarota, Ana Luizi Baracho, Livia Carneiro, Martín Cammarota

**Affiliations:** ^1^ Memory Research Laboratory—Brain Institute, UFRN Natal Rio Grande do Norte Brazil

**Keywords:** cannabinoid type‐1 receptors, hippocampus, object recognition memory, β‐adrenergic receptors

## Abstract

Object recognition memory (ORM) plays a key role in identifying familiar items and encoding episodic information. ORM consolidation depends on β‐adrenergic receptor (βAR) signaling and is associated with increased BDNF expression in the dorsal hippocampus. Although hippocampal activation of cannabinoid type‐1 receptors (CB1Rs) is known to impair ORM consolidation, the mechanisms underlying this effect remain unclear. In this study, we used the novel object recognition task to examine the interaction between CB1Rs and βARs during ORM consolidation in adult male Wistar rats. Intra‐dorsal CA1 infusion of the CB1R agonist ACEA, the βAR antagonist propranolol, or the PKA inhibitor myristoylated PKI_14–22_, administered 5‐min post‐training, impaired ORM consolidation. Notably, co‐administration of the PKA activator 8Br‐cAMP or the βAR agonist isoproterenol reversed ACEA‐induced amnesia. In contrast, the CB1R inverse agonist AM251 failed to reverse propranolol‐induced amnesia, which was instead rescued by recombinant BDNF infusion into the hippocampus 120‐min post‐training. These findings suggest that hippocampal CB1Rs regulate ORM consolidation by acting upstream of βARs via a signaling cascade involving PKA activation and BDNF expression.

Object recognition memory (ORM) allows animals to discriminate between familiar and novel objects, supporting episodic memory encoding and recall. Although ORM consolidation has traditionally been attributed to the perirhinal and prefrontal cortices, regions linked to familiarity‐based recognition and exploratory decision‐making, respectively, evidence increasingly implicates the hippocampus as a critical contributor, even in the absence of spatial or contextual demands (Cohen et al. 2013). This is supported by the association of ORM with CA3‐CA1 long‐term potentiation (LTP) and the activation of signaling cascades in dorsal CA1 (Clarke et al. 2010; Trimper et al. 2014; ILL‐Raga et al. 2013; Rossato, Gonzalez, Apolinário, et al. 2025).

Cannabinoid type 1 receptors (CB1Rs) are abundantly expressed in the hippocampus, mainly at presynaptic terminals (Mackie 2008). As key elements of the endocannabinoid system, CB1Rs regulate neural oscillations and synaptic plasticity (Robbe et al. 2006; Fontaine et al. 2020), both essential for memory consolidation. Indeed, CB1R activation has been shown to impair memory, including ORM (Yim et al. 2008; Clarke et al. 2008; Stern et al. 2017; Lisboa et al. 2024), indicating that CB1Rs inhibit consolidation. However, the molecular mechanisms underlying this impairment remain poorly understood.

Conversely, β‐adrenergic receptors (βARs) are well‐established facilitators of memory consolidation, acting mainly through PKA‐dependent activation of CREB‐driven gene transcription and subsequent protein synthesis (Tully and Bolshakov 2010). In the hippocampus, βAR activation after novel object exposure triggers cAMP/PKA signaling and upregulates memory‐related proteins, including BDNF (Kobayashi and Yasoshima 2001; Haenisch et al. 2008). Notably, ORM consolidation specifically depends on βAR activity, which is modulated upstream by nitric oxide (NO) and soluble guanyl cyclase (sGC) signaling (Furini et al. 2010). Since CB1Rs influence both NO synthase and sGC (Jones et al. 2008), and regulate noradrenaline release in multiple brain circuits (Hardingham et al. 2013; Carvalho and Van Bockstaele 2012), they may act upstream of hippocampal βARs, potentially shaping the molecular cascade required for ORM consolidation. Indeed, systemic CB1R antagonism enhances declarative‐like memory in a βAR‐dependent manner (Martinez‐Torres et al. 2023). However, direct evidence for hippocampal CB1R‐βAR interaction in ORM consolidation is lacking.

In this study, we tested the hypothesis that hippocampal CB1Rs regulate ORM consolidation via upstream modulation of βAR signaling in adult male Wistar rats. After a 4‐days habituation period to the training arena, a uniformly painted 60 × 60 × 60 cm gray plywood open‐field arena devoid of spatial or contextual cues, rats were trained in the novel object recognition (NOR) task, which capitalizes on rats' innate preference for novel objects (Ennaceur and Delacour 1988). During training, animals freely explored two different but behaviorally equivalent novel objects (objects A and B) for 5 min. These objects, made of metal, glass, or glazed ceramic, had no inherent significance. Control animals were exposed to the arena without objects.

ORM retention was assessed by re‐exposing animals to the arena containing one of the objects from training (object A) alongside a behaviorally equivalent novel object (object C) for 5‐min. Behavior was recorded using overhead video cameras. Video data were captured at 30 frames per second and analyzed using the ObjectScan system (CleverSys). Exploration was defined as sniffing or touching the objects with the muzzle or forepaws, while behaviors such as sitting on or turning around the objects were not considered exploratory. A discrimination index (DI) was calculated as follows: (Time exploring novel object−Time exploring familiar object)/Total object exploration time. DI scores range from −1 to +1, where positive values show a preference for the novel object, and values near zero indicate no discrimination between objects. NOR training resulted in ORM retention for at least 7‐days (Figure 1A; two‐way ANOVA: Test Day, *F*(2,22) = 12.21, *p* = 0.0003; Training Session, *F*(1,11) = 19.64, *p* = 0.001; Interaction, *F*(2,22) = 13.21, *p* = 0.0002). Object equivalence was confirmed in untrained animals (Figure 1B).

**FIGURE 1:**
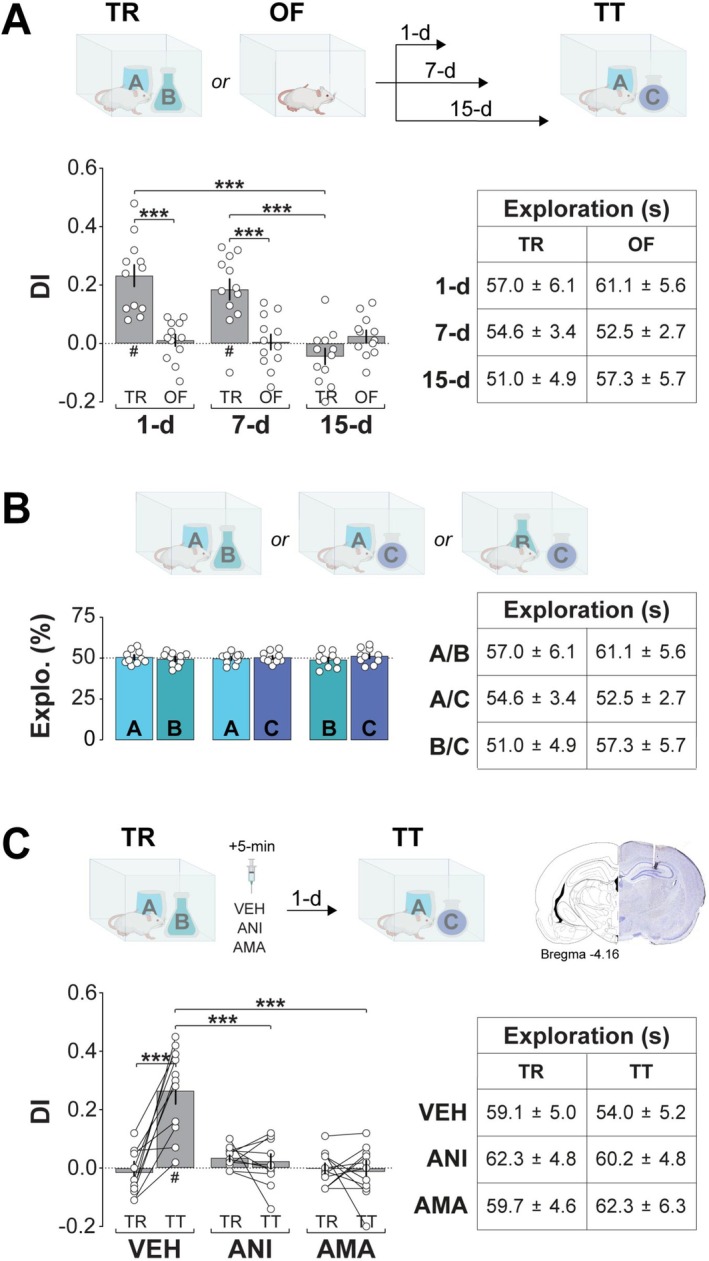
NOR training induces long‐lasting ORM that requires hippocampal protein synthesis and gene transcription for consolidation. (a) Top panel: Experimental timeline. Naïve adult male Wistar rats were habituated to the training arena without objects for 5‐min per day over four consecutive days. One day after the final habituation session, animals were either trained (TR) in the NOR task with two different, but behaviorally equivalent objects (A and B) or underwent an open field exploration session without objects (OF). Long‐term ORM retention was assessed in a 5‐min test session (TT) on days 1, 7, or 15 post‐training with object A and a novel object C. Bottom left panel: Discrimination index (DI) during TT for TR and OF animals. Bottom right panel: Total exploration time during TT. (b) Top panel: Habituated naïve adult male Wistar rats were exposed for 5‐min to two different objects in the training arena as indicated. Bottom left panel: Percentage of total exploration time spent on each object. Bottom right panel: Total exploration time. (c) Top left panel: Habituated naïve rats implanted with bilateral infusion cannulas targeting the dorsal CA1 region were trained (TR) in the NOR task with objects A and B, and 5‐min post‐training received intra‐CA1 infusions of vehicle (VEH; 0.1% DMSO in sterile saline), the protein synthesis inhibitor anisomycin (ANI), or the RNA polymerase II inhibitor α‐amanitin (AMA). ORM retention was assessed 1‐day later (TT) with familiar object A and novel object C. Top right panel: Representative photomicrograph showing histological verification of cannula placement in dorsal CA1. Bottom left panel: Discrimination index (DI) during TR and TT. Bottom right panel: Total exploration time during TR and TT. Discrimination indices (DIs) and exploration times are expressed as mean ± SEM (*n* = 10–12 per group). Dashed lines represent chance performance. Statistical significance: #*p* < 0.05 in one‐sample *t*‐test versus theoretical mean of 0, and ***p < 0.001 in Bonferroni's post hoc test after two‐way ANOVA. Normality was assessed using D’Agostino‐Pearson or Shapiro–Wilk tests.

For pharmacological manipulations, rats were implanted with bilateral 22‐gauge stainless steel guide cannulas targeting dorsal CA1 (coordinates: AP −4.2; LL, ±3.0; DV, −2.0; Paxinos and Watson 2007). Surgeries were performed under ketamine (80 mg/kg) and xylazine (10 mg/kg) anesthesia. Implants were secured to the skull with dental resin; animals received meloxicam (0.2 mg/kg) postoperatively and were allowed to recover for 7 days. All procedures followed NIH and ARRIVE guidelines and were approved by the local ethics committee (Comissão de Ética no Uso de Animais, Universidade Federal do Rio Grande do Norte). After the experiment, infusion sites were verified by injecting 4% methylene blue (1 μL) into dorsal CA1. A total of 345 animals were used in the experiments. Ten animals with misplaced cannula implants were excluded from analysis.

Intra‐CA1 infusion of the protein synthesis blocker anisomycin (100 μg/side) or the RNA polymerase II inhibitor α‐amanitin (45 ng/side) 5‐min post‐training impaired ORM when tested 1‐day later (Figure 1C; mixed‐model two‐way ANOVA: Session, *F*(1,30) = 16.28, *p* = 0.0003; Treatment, *F*(2,30) = 12.89, *p* < 0.0001; Interaction, *F*(2,30) = 20.62, *p* < 0.0001), confirming that ORM consolidation requires hippocampal gene transcription and protein synthesis (Rossato et al. 2007).

To explore CB1R and βAR involvement, rats received intra‐CA1 infusions of selective agents 5‐min post‐training. ACEA (0.40 fg/side), a CB1R agonist, significantly impaired ORM (Figure 2A; Session, *F*(1,16) = 4.56, *p* = 0.0485; Treatment, *F*(1,16) = 12.75, *p* = 0.0026; Interaction, *F*(1,16) = 15.83, *p* = 0.0011), consistent with prior findings (Clarke et al. 2008). ORM was similarly disrupted by the βAR antagonist propranolol (5 μg/side, Figure 2B; Session, *F*(1,18) = 10.63, *p* = 0.0043; Treatment, *F*(1,18) = 62.75, *p* < 0.0001; Interaction, *F*(1,18) = 5.271, *p* = 0.0339) or myristoylated PKI_14–22_ (4 μg/side; Figure 2C; mixed model two‐way ANOVA: Session, *F*(1,15) = 14.51, *p* = 0.0017; Treatment, *F*(1,15) = 13.87, *p* = 0.002; Interaction, *F*(1,15) = 9.347, *p* = 0.008), a specific cell‐permeable PKA inhibitor (Ma et al. 2009). These data implicate the CB1R/βAR/PKA axis in ORM consolidation.

**FIGURE 2:**
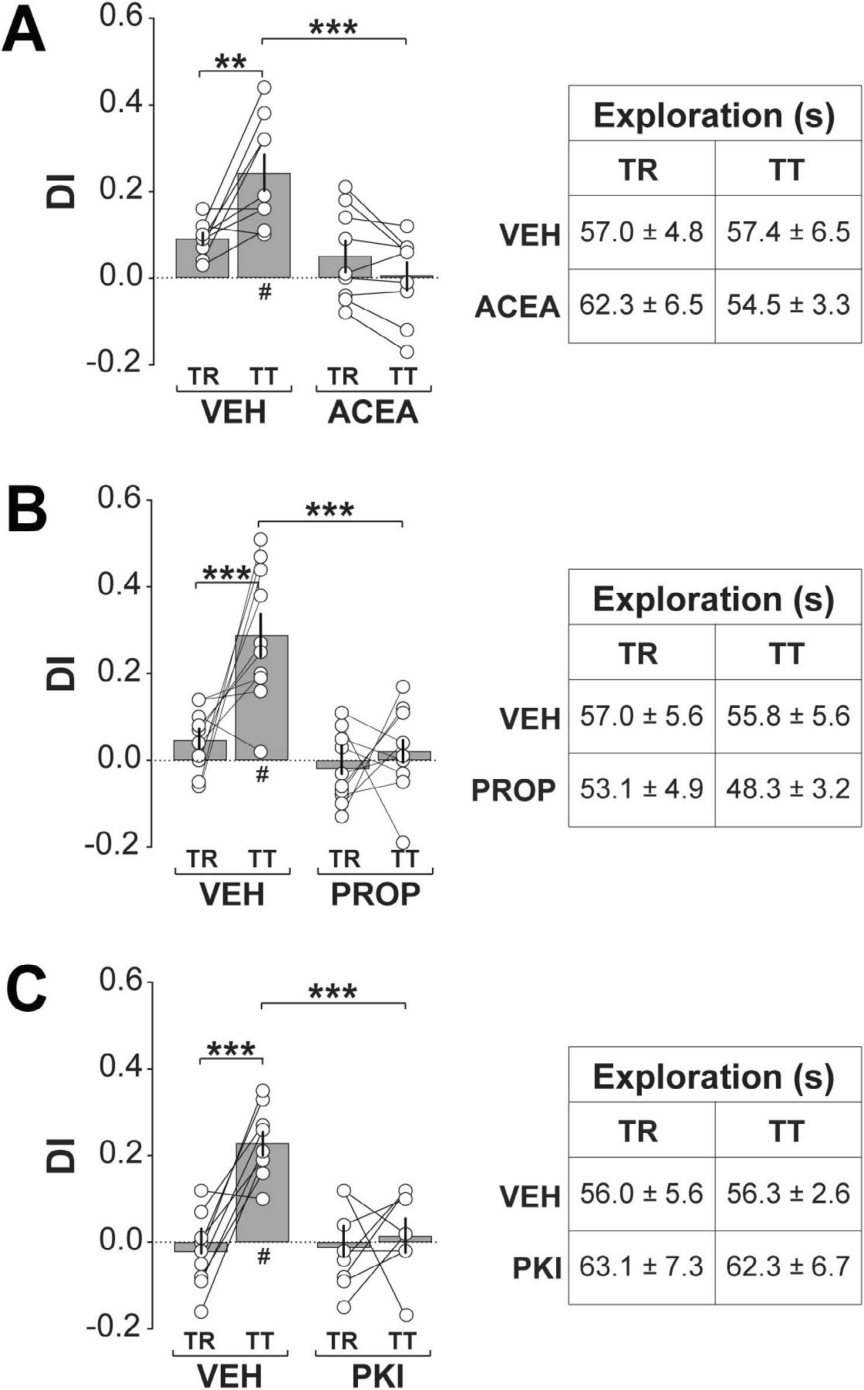
Post‐training CB1R activation, βAR blockade, or PKA inhibition impairs ORM consolidation. (a) Naïve adult male Wistar rats implanted with bilateral infusion cannulas targeting the dorsal CA1 region were habituated to the training arena without objects for 5‐min per day over four consecutive days. One day after the final habituation session, animals were trained (TR) in the NOR task with two different, but behaviorally equivalent objects (A and B), and 5‐min post‐training received intra‐CA1 infusions of vehicle (VEH; 0.1% DMSO in sterile saline), or the CB1R agonist ACEA. ORM retention was assessed 1‐day later (TT) with familiar object A and novel object C. Left panel: Discrimination index (DI) during TR and TT. Right panel: Total exploration time during TR and TT. (b) Animals were trained, treated, and tested as in (a) but received VEH or the βAR antagonist propranolol (PROP) in dorsal CA1. (c) Animals were trained, treated, and tested as in (a) but received VEH or the PKA inhibitor myristoylated PKI_14–22_ (PKI) in dorsal CA1. Discrimination indices (DIs) and exploration times are expressed as mean ± SEM (*n* = 8–10 per group). Dashed lines represent chance performance. Statistical significance: #*p* < 0.05 in one‐sample t‐test vs. theoretical mean of 0. **p < 0.01 and ***p < 0.001 in Bonferroni's post hoc test after two‐way ANOVA. Normality was assessed using D’Agostino‐Pearson or Shapiro–Wilk tests.

To determine whether enhancing βAR signaling could rescue CB1R‐induced deficits, we co‐infused ACEA with either the phosphodiesterase‐resistant PKA activator 8‐Br‐cAMP (1.25 μg/side; Figure 3A) or the βAR agonist isoproterenol (1.25 μg/side; Figure 3B). Both treatments fully restored ORM (8‐Br‐cAMP: Session, *F*(1,37) = 94.2, *p* < 0.0001; Treatment, *F*(3,37) = 4.03, *p* = 0.0141; Interaction: *F*(3,37) = 8.401, *p* = 0.0002. Isoproterenol: Session, *F*(1,28) = 45.65, *p* < 0.0001; Treatment, *F*(3,28) = 4.916, *p* = 0.0072; Interaction: *F*(3,28) = 4.159, *p* = 0.0148). Together, these results support a model in which CB1R activation impairs ORM by dampening noradrenergic activation and βAR/PKA signaling.

**FIGURE 3:**
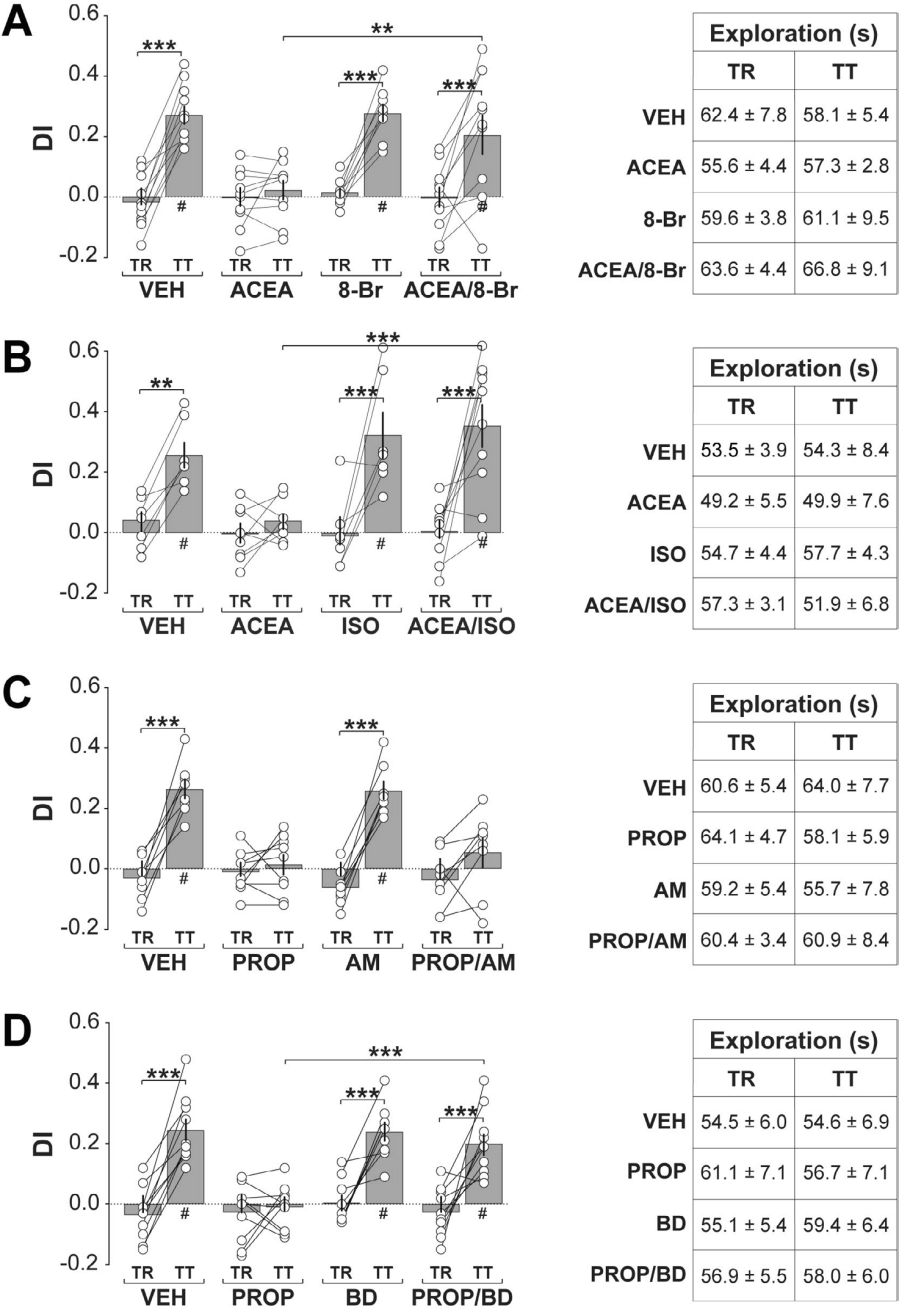
CB1R‐induced amnesia is rescued by PKA activation or βAR agonism, whereas βAR blockade‐induced amnesia is not reversed by CB1R antagonism but is rescued by BDNF. (a) Naïve adult male Wistar rats implanted with bilateral infusion cannulas targeting the dorsal CA1 region were habituated to the training arena without objects for 5‐min per day over four consecutive days. One day after the final habituation session, animals were trained (TR) in the NOR task with two different, but behaviorally equivalent objects (A and B), and 5‐min post‐training received intra‐CA1 infusions of vehicle (VEH; 0.1% DMSO in sterile saline), the CB1R agonist ACEA, the PKA activator 8‐Br‐cAMP (8‐Br), or ACEA plus 8‐Br‐cAMP (ACEA/8‐Br). ORM retention was assessed 1‐day later (TT) with familiar object A and novel object C. Left panel: Discrimination index (DI) during TR and TT. Right panel: Total exploration time during TR and TT. (b) Animals were trained, treated, and tested as in (a) but received ACEA, the βAR agonist isoproterenol (ISO), or ACEA plus isoproterenol (ACEA/ISO) in dorsal CA1. (c) Animals were trained, treated, and tested exactly as in (a) but received the βAR antagonist propranolol (PROP), the CB1R inverse agonist AM251 (AM), or propranolol plus AM251 (PROP/AM) in dorsal CA1. (d) Animals were trained, treated, and tested as in (a) but received PROP 5‐min post‐training, BDNF (BD) 120‐min post‐training, or PROP 5‐min post‐training plus BDNF 120‐min post‐training (PROP/BD) in dorsal CA1. Discrimination indices (DIs) and exploration times are expressed as mean ± SEM (*n* = 7–11 per group). Dashed lines represent chance performance. Statistical significance: #*p* < 0.05 in one‐sample *t*‐test versus theoretical mean of 0. **p < 0.01 and ***p < 0.001 in Bonferroni's post hoc test after two‐way ANOVA. Normality was assessed using D’Agostino‐Pearson or Shapiro–Wilk tests.

To further examine pathway hierarchy, we co‐infused propranolol with the CB1R antagonist/inverse agonist AM‐251 (300 ng/side), which facilitates neurotransmitter release, potentiates the promnesic effect of noradrenaline on contextual fear memory, and enhances ORM when administered systemically (Kreitzer and Regehr 2001; Pertwee 2005; Bialuk and Winnicka 2011; Gazarini et al. 2022). AM‐251 failed to reverse propranolol‐induced amnesia (Figure 3C; Session, *F*(1,29) = 92.33, *p* < 0.0001; Treatment, *F*(3,29) = 5.722, *p* = 0.0033; Interaction, *F*(3,29) = 15.12, *p* < 0.0001), indicating that βARs operate downstream of CB1Rs in this signaling cascade. In contrast, intra‐CA1 infusion of recombinant BDNF (0.25 μg/side) 120‐min after propranolol administration, coinciding with the βAR‐dependent peak in hippocampal BDNF levels during ORM consolidation (Furini et al. 2010), fully restored ORM (Figure 3D; Session, *F*(1,35) = 80.31, *p* < 0.0001; Treatment, *F*(3,35) = 9.995, *p* < 0.0001; Interaction, *F*(3,35) = 7.79, *p* = 0.0004), implicating BDNF as a key downstream effector in the CB1R–βAR–PKA pathway.

CB1Rs primarily couple to Gi/o protein to inhibit presynaptic neurotransmitter release (Araque et al. 2017), thereby regulating synaptic activity and maintaining homeostatic balance. This inhibitory influence may also serve to prevent over‐consolidation of memory traces (Lupica et al. 2004), a mechanism particularly relevant in memory systems requiring adaptive flexibility, such as ORM. Our results endorse this assertion, revealing that CB1Rs modulate ORM consolidation by suppressing βARs signaling, likely by controlling hippocampal noradrenergic activation. Specifically, CB1R activation impaired ORM via a mechanism that was reversed by either direct activation of βARs or downstream PKA signaling, which places CB1Rs functionally upstream of βARs in the molecular cascade that supports ORM. This hierarchical relationship is further supported by our observation that CB1R antagonism could not reverse the amnesia induced by βAR, whereas delayed BDNF infusion, timed to coincide with peak βAR‐dependent BDNF expression, successfully restored memory. This highlights BDNF as a key downstream effector of the CB1R/βAR/PKA signaling axis, aligning with previous studies showing that post‐training BDNF administration can rescue LTP and memory even when brain protein synthesis, a hallmark of memory consolidation, is inhibited. This regulatory mechanism may influence excitation‐spike coupling via βARs (Bacon et al. 2020), facilitate synaptic potentiation (Cohen et al. 2017), and promote BDNF‐dependent gene expression (Furini et al. 2010), which, consistent with its role in other memory types, appears to be an essential mediator of CB1R‐regulated, βAR‐dependent ORM consolidation. In line with this hypothesis, prior studies have shown that CB1R activation abolishes both electrically and pharmacologically evoked noradrenaline release in hippocampal slices, an effect that is blocked by the CB1R antagonist SR141716 (Schlicker et al. 1997; Kathmann et al. 1999). Moreover, CB1Rs modulate BDNF levels in a dynamic, often reciprocal manner (Maj et al. 2007). This interaction may represent a bidirectional feedback loop wherein CB1Rs constrain βAR‐driven memory enhancement, while βARs activation counteracts CB1R‐induced memory suppression (Navarro et al. 2012). Such modulation may be clinically relevant, particularly in Alzheimer's disease, where CB1R dysregulation contributes to cognitive deficits (Manuel et al. 2014). Accordingly, targeting βAR signaling to rebalance this system might offer a promising therapeutic avenue (Gannon et al. 2015).

Further research should investigate other signaling systems that may converge on this pathway during ORM consolidation. The NO/cGMP/PKG cascade, a known modulator of βAR/PKA‐dependent plasticity (Bollen et al. 2014), and the cholinergic system, which contributes to ORM consolidation (De Jaeger et al. 2013) and interacts with both β‐adrenergic and endocannabinoid networks during memory processing (de Leo et al. 2022; Moreno‐Rodríguez et al. 2025), are promising candidates. Indeed, CB1Rs engage in complex reciprocal crosstalk with both pathways in a manner that may be either synergistic or antagonistic depending on specific molecular factors and behavioral context that are still poorly understood (Tzavara et al. 2003; From and Crosby 2025).

Although several studies have reported ORM deficits following hippocampal lesions (Broadbent et al. 2010; Lacoursiere et al. 2023), others have not (Mumby et al. 2002; Ainge et al. 2006), leading some researchers to suggest that the hippocampus may primarily support the spatial or contextual components of the task, rather than memory consolidation itself (Barker and Warburton 2011). However, substantial evidence now points to a more direct role for the hippocampus in ORM. The conflicting findings likely reflect methodological differences, including variations in task design, species or strain characteristics, object features, and the nature and amount of contextual information provided during habituation, training, and testing (Cohen and Stackman Jr 2015).

Recent research underscores the hippocampus' essential role not only in ORM consolidation (Cohen et al. 2013; Stackman Jr et al. 2016; Tanimizu et al. 2018), but also in ORM recall and reconsolidation (Suárez‐Pereira and Carrión 2015; Radiske et al. 2017; Rossato et al. 2019, 2023; Rossato, Gonzalez, Souza, et al. 2025; Gonzalez et al. 2019; Gonzalez, Radiske, et al. 2021; Gonzalez, Rossato, et al. 2021), even when spatial and contextual cues are minimized. This is particularly evident in NOR protocols using pre‐validated, behaviorally equivalent objects arranged symmetrically within a uniformly painted familiar arena, as in the present experiments. Under these controlled conditions, several hippocampal signaling pathways have been shown to contribute to ORM consolidation, including JNK, estrogen receptor α, histone acetyltransferase, mTOR, and nociceptin/orphanin FQ (Goeldner et al. 2008; Myskiw et al. 2008; Zhao et al. 2012; Boulware et al. 2013; Rossato et al. 2022).

Complementary evidence from molecular, chemogenetic, electrophysiological, and pharmacological studies further supports this view. For example, knockdown of adult neurogenesis in the dentate gyrus impairs ORM (Jessberger et al. 2009), reversible inactivation of the dorsal hippocampus disrupts consolidation (Hammond et al. 2004; Tuscher et al. 2018; Cinalli Jr et al. 2020), and interference with hippocampal theta rhythms induces object‐specific amnesia (Gonzalez et al. 2022). Additionally, NOR training is accompanied by hippocampal acetylcholine efflux (Stanley et al. 2012) and LTP‐like enhancements at both glutamatergic and GirK‐dependent components of the dorsal CA3–CA1 synapse (Sánchez‐Rodríguez et al. 2022). Together, these findings establish the hippocampus as a key site for ORM consolidation and frame the CB1R‐ and βAR‐dependent mechanisms described here.

Finally, we acknowledge the exclusive use of male rats as a limitation. Given documented sex differences in cannabinoid and βAR signaling, exploring these dynamics in females will be a priority for future studies.

## Funding

This work was supported by the Conselho Nacional de Desenvolvimento Científico e Tecnológico (407459/2021‐9, 307433/2022‐6) and the Coordenação de Aperfeiçoamento de Pessoal de Nível Superior (001).

## Conflicts of Interest

The authors declare no conflicts of interest.

## Data Availability

The data that support the findings of this study are available from the corresponding author upon reasonable request.
